# Integrated Phytochemical Characterisation, Pharmacological Evaluation and Computational Investigation of *Acampe papillosa* Stem Extract

**DOI:** 10.1002/ansa.70097

**Published:** 2026-07-23

**Authors:** Qurratul Ain Sadia, Sadia Tamanna Tamim, Faruq Mohammed Tashriq, Nasir Uddin, Fatematus Zhohara Alam Hima, Ulfat Sobha Surat, Mily Khastagir, Muhammad Abdul Jalil, Md. Hossain Rasel, Arman Ullah Rafi, S. M. Moazzem Hossen

**Affiliations:** ^1^ Department of Pharmacy, Faculty of Biological Sciences University of Chittagong Chattogram Bangladesh; ^2^ Department of Pharmacy BGC Trust University Bangladesh Chattogram Bangladesh

**Keywords:** *Acampe papillosa*, fever, in silico, neuropharmacology, orchid, pain

## Abstract

*Acampe papillosa*, an orchid species traditionally utilised as an herbal medicine, has yet to be extensively researched for its potential pharmacological properties. This study examined the potential for anti‐anxiety, sedative, anti‐inflammatory and antipyretic effects of methanol extract of the stem of *A. papillosa* (APSME) through in vivo and in silico approaches. A dose of 200 and 400 mg/kg was administered to Swiss albino rodents. When examined in the elevated plus maze, APSME‐treated mice spent significantly longer periods of time in the open arms of the maze at both dosages than control animals (*p* < 0.001), indicating an anxiolytic effect of APSME. The sedative effects of APSME were assessed by measuring locomotor activity using an open field test and a hole cross test, with both methods showing a significant decrease in locomotion as APSME doses increased from 200 to 400 mg. Following treatment of rodents with carrageenan‐induced paw oedema using APSME (400 mg/kg), significant reductions in the thickness of the paw were observed at 4 h post‐administration compared to control groups (*p* < 0.001), indicating strong anti‐inflammatory activity. Antipyretic effects of APSME were measured using a brewer's yeast‐induced pyrexia animal model and were accompanied by statistically significant reductions in rectal temperature at 21 h post‐treatment (98.04 ± 0.05°F). Molecular docking and PASS prediction further support these findings by showing favourable interactions between the plant phytochemicals and relevant biological targets. Overall, the results suggest that *A. papillosa* could be a promising natural source for developing treatments for anxiety, inflammation, fever and related conditions.

## Graphical abstract

The pharmacological potential of the methanolic stem extract of Acampe papillosa is evaluated using in vivo and in silico approaches. The extract demonstrates significant anxiolytic, sedative, anti‐inflammatory, and antipyretic activities in experimental models, while molecular docking supports the observed biological effects through favorable interactions between phytochemicals and therapeutic targets.

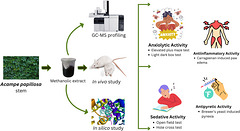

## 1 Introduction

Among the most prevalent neuropsychiatric problems in the world, anxiety disorders have a substantial impact on social, vocational and psychological functioning. Despite the therapeutic efficacy of traditional anxiolytic medications like benzodiazepines, extended use of these medications is frequently linked to side effects such as dependency, drowsiness, tolerance and withdrawal symptoms [1]. Many people have waxing and waning generalised anxiety disorder, with significant depressive episodes occurring at times of extreme stress. This combination of severe depression and generalised anxiety disorder is known as “anxious depression,” and it is a specific, prevalent condition in primary care [2]. Being female, having a low socioeconomic background and experiencing childhood trauma—such as physical or sexual abuse, neglect, exposure to parental violence, alcoholism, or drug misuse—are recognised risk factors for developing generalised anxiety disorder [3]. Recent studies indicate that experiencing physical punishment during childhood may raise the likelihood of developing generalised anxiety disorder later in adulthood [4]. These risk causes, however, are general and may also raise the likelihood of developing other mood and anxiety disorders [5].

Elderly people's quality of life is frequently impacted by insomnia [6]. Drugs are often used to treat acute gout [7]. Sedative‐related side effects, including ataxia, falls, or memory loss, are believed to be especially harmful to the elderly [8, 9].

Inflammation is the body's protective response to infection or tissue injury, playing a vital role in survival. Normal tissue homeostasis depends on inflammatory responses. Finding specific molecular patterns associated with infection or tissue damage is the first step in the intricate process of the molecular mechanism of inflammation. Many important regulators involved in the selective synthesis of proinflammatory chemicals regulate the overall inflammatory response. Prolonged inflammation is often linked to serious adverse health outcomes [10]. Inflammatory illnesses, such as various types of rheumatic diseases, are a leading worldwide health matter. The majority of people suffer from inflammatory disorders. Although many medications are available for the treatment of inflammatory disorders, prolonged use of these drugs may lead to serious adverse effects. In conventional therapy, both steroidal and non‐steroidal agents are commonly used to manage inflammation. Non‐steroidal anti‐inflammatory drugs (NSAIDs) work by inhibiting cyclooxygenase (COX), thereby preventing the early formation of prostaglandins. As a result, NSAIDs play an important role in reducing the damaging effects associated with inflammation [11].

Fever is a regulated rise in body temperature caused by an increase in the hypothalamic temperature set point. The hypothalamus stimulates physiological and behavioural processes that promote heat generation and retention until a freshly increased set point temperature is reached [12]. The immune response carefully controls fever. Endogenous antipyretic compounds are released in response to inflammatory stimuli that cause porphyritic signals to be generated [13]. Pyrexia can be controlled both centrally and peripherally by drugs including glucocorticoids, arginine vasopressin and α‐melanocyte‐stimulating hormone. Fever suppression is one of the many anti‐inflammatory characteristics of the cytokine interleukin. Furthermore, certain cytochrome P‐450 enzymes produce a type of lipid molecule called epoxy eicosanoids, which are crucial in reducing inflammation and fever [14, 15].

A well‐established computational method for predicting the vitality of cooperation between two particles is in silico molecular docking. This system primarily uses algorithms such as fragment‐based methodology, stimulation and molecular dynamics [16]. Molecular docking studies are utilised to locate the optimal ligand introduction that would create a complex with the least amount of overall energy and to ascertain how two particles communicate [17].

The use of plants, plant extracts and naturally derived compounds for disease treatment is a long‐standing therapeutic approach, even though much of the scientific evidence supporting these therapies is still developing. In fact, a natural product prototype is currently available for many pharmacological families of drugs. Medicinal plants have historically generated a variety of chemicals, including quinine, atropine, reserpine, morphine, digoxin, physostigmine, vinblastine, pilocarpine, vincristine, taxol and artemisinin [18]. In the primary health care systems of communities with limited resources, traditional medicine has traditionally been the most affordable and readily available therapeutic option. Plants have traditionally been used medicinally by the indigenous. Medicinal plants have been used for therapeutic purposes since ancient times. The scriptures claim that plants have been used medicinally from 4000–5000 B.C., with the Chinese being the first to employ natural herbal remedies [19]. The epiphytic orchid species *A. papillosa* has the potential to be decorative due to its multiflowered, pendulous racemes with yellow‐green blooms and its evergreen, clustered foliage. Its medicinal use is also well‐established; rheumatism is treated with its roots [20]. Due to habitat degradation and commercial collecting, its native numbers are declining. However, the procedures for its widespread dissemination have not yet been established [21]. An indigenous orchid from Bangladesh called lindl. has been used to treat male and female problems, fever, earaches and injuries [22] as well as rheumatism, sciatica and neuralgia [23]. The phytochemicals and neuropharmacological properties of certain orchid species are investigated in many studies [24]. Previous studies on the constituents of *A. papillosa* have identified several chemical compounds, such as *N*‐methyl‐4‐pyridinamine, dimethyl sulfone, p‐cresol, ethenone, 1‐(2,4,6‐trihydroxyphenyl), benzene acetic acid, 2‐phenylethyl ester, 2‐butyn‐1‐al diethyl acetal, 6‐ethyl‐5,6‐dihydro‐2H‐pyran‐2‐one, 1,3,5‐cycloheptatriene, 7‐ethyl, phenol, 4‐(methoxymethyl), ethenone, 1‐(2‐hydroxy‐5‐methylphenyl), 1‐(4‐Hydroxyphenyl)propane‐1,2‐diol, homovanillyl alcohol, 2,4‐di‐*tert*‐butylphenol, methyl 3‐(4‐hydroxy‐3‐methoxyphenyl)propanoate and so on. The orchid extract was found to have antibacterial, cytotoxic, analgesic and anxiolytic properties [24]. Therefore, this study aimed to investigate the bioactive phytochemicals present in the orchid and evaluate the anxiolytic, sedative, anti‐inflammatory and antipyretic activities of the methanolic extract of *A. papillosa* stem (APSME) using both in vivo and in silico approaches.

## 2 Materials and Methods

### 2.1 Plant Collection and Identification

*A. papillosa* was collected from Bandarban, Bangladesh, in April 2025. The plant was authenticated by Shaikh Bokhtear Uddin. The stems of *A. papillosa* were then separated for further analysis.

### 2.2 Drugs and Chemicals

Standard medications for various tests were bought from the pharmacy, such as diazepam for sedative and anxiolytic activity tests, diclofenac for anti‐inflammatory activity tests and paracetamol for antipyretic activity tests. Reagents and chemicals of analytical grade were utilised. For control, we have used a Tween‐80 solution.

### 2.3 Preparation of Stem Extract

Fresh *A. papillosa* stems were collected, washed with distilled water, cut into small pieces and air‐dried at room temperature for approximately 20 days. The dried stems were then powdered and stored in an airtight container. About 790 g of the powdered material was macerated in 8 L of 99% methanol for 7 days with occasional stirring. The extract was filtered using Whatman No. 1 filter paper and concentrated under reduced pressure below 50°C using a rotary vacuum evaporator. The concentrated extract was then air‐dried to remove residual solvent completely. After repeating the process three times, 15 g of greenish stem extract (1.9% w/w) was obtained and stored at 4°C for further use [25].

### 2.4 Identification of Compounds by Gas Chromatography–Mass Spectrometry

A Shimadzu GC‐17A gas chromatograph and an MS TQ 8040 mass spectrometer were used for the gas chromatography–mass spectrometry (GC‐MS) analyses. The material was separated using Rxi‐5 MS (0.25 mm × 30 m) and DB1 (J & W) columns. The carrier gas employed was helium at a rate of 0.6 mL/min. The study circumstances comprised an input temperature of 260°C, an interfacing temperature of 280°C and an oven temperature of 70°C that ramped up to 150°C at 10°C/s during the first 10 min [26].

### 2.5 Experimental Animal

The animals were separated into four groups: control, standard and two treatment groups. The control group was given 1% Tween 80, while the standard groups received diazepam (1 mg/kg), indomethacin (10 mg/kg), or paracetamol (150 mg/kg). The treatment groups were administered the extract at doses of 200 and 400 mg/kg to assess dose‐related effects [27].

### 2.6 Acute Toxicity Study

An established approach was used to evaluate the extract's acute toxicity. Following an overnight fast, groups of five mice were given dosages of 1000, 2000, 3000 and 4000 mg/kg of APSME. Food was withheld from the mice for 3–4 h following treatment. Daily, 24‐h and 30‐min observations were made. The mice were monitored for any alterations in neurological activity, respiratory and circulatory function, as well as changes in the eyes, fur, skin and mucous membranes. The median lethal dosage (LD50) is ten times the effective dose [28].

### 2.7 Experimental Design

The experimental animals were divided into four groups: a control group, a standard group, and two test groups. The control group received a 1% Tween 80 solution. Standard treatments included diazepam (1 mg/kg), indomethacin (10 mg/kg) and paracetamol (150 mg/kg). The dose‐dependent effects were evaluated using two test doses of 200 and 400 mg/kg.

### 2.8 Anxiolytic Profiling

#### 2.8.1 Elevated Plus Maze Test

The elevated plus maze (EPM) method was used to determine anti‐anxiety activity. The apparatus consisted of four arms—two open and two enclosed—positioned in a plus‐shaped design at a height of 50 cm from the ground. Animals were grouped and treated following the procedure described in Section 2.6. Each mouse was then observed for 5 min, and the time spent in the open and closed arms was recorded [29].

#### 2.8.2 Light‐Dark Box Test

Two wooden boxes measuring 45 × 27 × 27 cm^3^ that were fastened to one another were utilised in this test. One box was covered with plywood to make it gloomy, while the other was lit by a 60 W lamp that was suspended 25 cm above. The mice were allocated into groups and treated as described in Section 2.6. Each animal was individually placed at the centre of the light chamber and monitored for 5 min. The duration spent in both the light and dark compartments was then recorded [30].

### 2.9 Sedative Profiling

#### 2.9.1 Open Field Test

To evaluate sedative activity, a standardised method was followed. The mice were divided into four groups (*n* = 5), and each group received different doses of the extract as described in Section 2.6. Locomotor activity was then assessed by recording the number of square crossings made by each mouse for 3 min at 0, 30, 60, 90 and 120 min throughout the experiment [31].

#### 2.9.2 Hole Cross Test

A steel cage measuring 30 × 20 × 14 cm was used for the experiment, with a central partition containing an opening of 3 cm in diameter and 7.5 cm in height. Treatment was carried out according to Section 2.6. Each animal was placed inside the apparatus, and the number of crossings between the two chambers through the opening was recorded over 3 min at 0, 30, 60, 90 and 120 min [32].

### 2.10 Anti‐inflammatory Activity

#### 2.10.1 Carrageenan‐Induced Paw Oedema

The carrageenan‐induced acute inflammation model is a well‐established method for assessing the anti‐inflammatory activity of test compounds. In this study, carrageenan was used to induce oedema in the right hind paw of mice. Treatments were administered according to Section 2.6, with the test sample given 1 h prior to and the standard drug 30 min before carrageenan injection. A 100 µL dose of 1% (w/v) carrageenan was injected subcutaneously into the right hind paw. Paw circumference (mm) was recorded before induction and at 1, 2, 3 and 4 h after injection. Finally, the percentage inhibition of oedema was determined using the relevant equation [33].

$$\begin{array}{cc} \mathrm{Percent}\;\mathrm{inhibition}\; & =\frac{\left(\mathrm{Ct}\;-\;\mathrm{Co}\right)\mathrm{Control}\;-\;\left(\mathrm{Ct}\;-\;\mathrm{Co}\right)\mathrm{Treated}}{\left(\mathrm{Ct}\;-\;\mathrm{Co}\right)\mathrm{Control}}\;\\ & \;\times 100 \end{array}$$

Here, Ct represents the mean paw circumference of each group at a given time point, while Co denotes the mean paw circumference of each group before carrageenan administration.

### 2.11 Antipyretic Activity

#### 2.11.1 Brewer's Yeast‐Induced Pyrexia

Antipyretic activity was assessed using a brewer's yeast–induced pyrexia model. Fever was induced by subcutaneously injecting a 20% aqueous suspension of brewer's yeast at a dose of 10 mL/kg body weight. Prior to induction, the animals were fasted overnight while maintaining free access to water. Baseline rectal temperature (91.74 ± 0.72°F) was measured using an Ellab thermometer. After 18 h, animals showing a rise of 0.54–0.90°F in rectal temperature were selected for the experiment. Treatments were then given as outlined in Section 2.6, and rectal temperature was recorded hourly for 3 h following administration [34].

$$Percentage\;of\;reduction\;of\;pyrexia=\frac{B-C}{B-A}\;\times 100$$

Here, *A* = Normal body temperature; *B* = Rectal temperature at 24 h after yeast administration; *C* = Rectal temperature after drug administration at a different time interval.

### 2.12 In Silico Study

#### 2.12.1 Ligand Preparation

The compounds included in the final dataset were obtained in 3D SDF format from the PubChem database. For molecules lacking available 3D conformers in PubChem, Open Babel software was used to convert their 2D SDF structures into 3D SDF format. Before molecular docking studies, the ligand structures were energy‐minimised using PyRx software [35].

#### 2.12.2 Protein Preparation

For molecular docking studies related to anti‐inflammatory, anxiolytic, sedative and antipyretic activities, the target proteins Cyclooxygenase‐2 (PDB ID: 5KIR), Monoamine Oxidase A (PDB ID: 2Z5X), gamma‐aminobutyric acid (GABA)‐A receptor (PDB ID: 6HUP) and Microsomal Prostaglandin E Synthase‐1 (PDB ID: 3DWW) were retrieved in PDB format from the RCSB Protein Data Bank (https://www.rcsb.org/structure). The protein structures were prepared by removing water molecules and other heteroatoms using Discovery Studio 2020. Subsequently, energy minimisation was performed using both conjugate gradient and steepest descent algorithms in Swiss‐PDBViewer (version 4.1.0). Finally, the optimised proteins were converted into pdbqt format using AutoDock Tools (version 1.5.6) and stored for docking analysis [36].

#### 2.12.3 Tools for Software

The analysis was carried out using several computational tools and databases, including the Protein Data Bank (PDB), DrugBank, Swiss‐PDB Viewer, AutoDock Vina, MGL Tools, PubChem and Discovery Studio Visualizer 2021 (BIOVIA).

#### 2.12.4 Analysis of Molecular Docking

Docking calculations were performed using protein structures retrieved from the RCSB Protein Data Bank. The resulting docking poses were analysed using PyMOL (version 2.6.2), a powerful molecular visualisation and analysis tool. This approach enabled the identification of various interaction types contributing to ligand binding, including hydrogen bonds, π–π stacking and cation–π interactions. In addition, PyMOL provided detailed insights into ligand–receptor interactions, allowing a clearer understanding of the underlying binding mechanisms. This strategy is in line with recent developments in protein‐ligand docking, tackling both cutting‐edge techniques and upcoming difficulties in the area [37].

#### 2.12.5 PASS Prediction

The PASS online tool (http://www.pharmaexpert.ru/passonline/predict.php) was used to predict the potential biological activities of the selected compounds. The results are expressed as Pa (probability of activity) and Pi (probability of inactivity), ranging from 0.000 to 1.000. A compound is considered to have potential biological activity when its Pa value is higher than its Pi value. In general, strong pharmacological activity is indicated by Pa > 0.7, moderate activity is suggested when Pa is below 0.7, and weak activity is considered when Pa < 0.5 [38].

### 2.13 Statistical Analysis

All data are presented as mean ± standard error of the mean (SEM) from three independent replicates. Statistical analysis was performed using one‐way ANOVA followed by Dunnett's test for multiple comparisons, with a 95% confidence interval considered significant at *p* < 0.05. All analyses were conducted using GraphPad Prism software (version 9.5) [39].

## 3 Result

### 3.1 GC–MS Profiling

**FIGURE 1:**
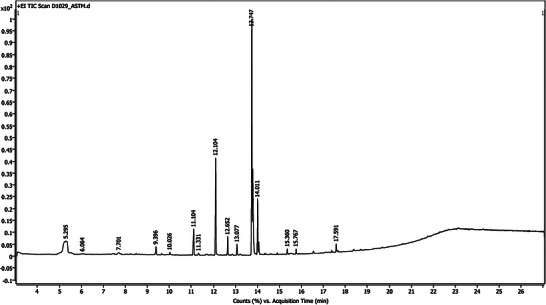
Gas chromatography–mass spectrometry (GC‐MS) chromatogram of the methanolic extract of *A. papillosa* stem.

26 compounds were identified in the methanol extract of *A. papillosa* using GC‐MS analysis. A reference chromatogram of GC‐MS analysis (the chromatograms in Figure 1 indicate the distinct characteristics of this chromatogram) is provided along with information on bioactive compounds: A list of bioactive compounds identified from APSME based on their retention time (RT), molecular formula, molecular weight and area is shown in Table 1. The mass spectra of each compound are provided in the Supporting Information.

**TABLE 1:** Phytochemical composition of the methanolic extract of *A. papillosa* stem.

Serial No.	Compound name	Molecular weight (g/mol)	RT (min)	Area
1	*N*‐Methyl‐4‐pyridinamine	108.14	3.3	571,468
2	Dimethyl sulfone	94.14	3.5	478,548
3	p‐Cresol	108.14	3.5	5,199,230
4	2‐Acetyl‐5‐methylfuran	124.14	3.7	1,302,261
5	Ethanone, 1‐(2,4,6‐trihydroxyphenyl)‐	168.15	4.0	18,147
6	Benzeneacetic acid, 2‐phenylethyl ester	240.3	4.8	15,894,287
7	2‐Butyn‐1‐al diethyl acetal	142.2	5.1	457,675
8	6‐Ethyl‐5,6‐dihydro‐2H‐pyran‐2‐one	126.15	5.2	21,603,063
9	1,3,5‐Cycloheptatriene, 7‐ethyl‐	120.19	5.2	33,093,663
10	Phenol, 4‐(methoxymethyl)‐	138.16	5.3	1,276,360,385
11	Ethanone, 1‐(2‐hydroxy‐5‐methylphenyl)‐	150.17	5.6	35,011,427
12	1‐(4‐Hydroxyphenyl)propane‐1,2‐diol	168.19	5.8	13,754,670
13	Homovanillyl alcohol	168.19	6.5	20,285,092
14	2,4‐Di‐*tert*‐butylphenol	206.32	7.7	160,997,120
15	Methyl 3‐(4‐hydroxy‐3‐methoxyphenyl)propanoate	210.23	9.7	63,721,675
16	Methyl tetradecanoate	242.4	10.0	78,813,268
17	Pentadecanoic acid, methyl ester	256.42	11.1	427,653,607
18	Hexadecanoic acid, methyl ester	270.5	12.1	3,597,710,935
19	Heptadecanoic acid, methyl ester	284.5	13.1	330,766,308
20	9,12‐Octadecadienoic acid (Z,Z)‐, methyl ester	294.5	13.7	7,985,538,490
21	11‐Octadecenoic acid, methyl ester	296.5	13.8	2,324,215,768
22	9‐Octadecenoic acid (Z)‐, methyl ester	296.5	13.8	141,678,634
23	Methyl stearate	298.5	14.0	1,853,810,645
24	Nonadecanoic acid, methyl ester	312.5	14.9	50,744,463
25	Eicosanoic acid, methyl ester	326.6	15.8	155,516,980
26	Docosanoic acid, methyl ester	354.6	17.4	67,504,863

### 3.2 Anxiolytic Activity

#### 3.2.1 EPM Test

According to the EPM test, APSME at doses of 200 and 400 mg/kg significantly increased the time spent in the open arms, indicating anxiolytic activity. The extract showed a highly significant effect (*p* < 0.001) at both doses compared to the control group, with outcomes comparable to those observed with the standard drug diazepam (Table 2).

**TABLE 2:** Effects of the methanolic extract of *A. papillosa* stem on the Elevated Plus Maze Test.

Group	Time spent in open arm	No of entries in open arms	Time spent in closed arm	Number entries in closed arm
**Control**	9.24 ± 1.15	2.20 ± 0.37	291.0 ± 1.7	17.2 ± 2.0
**Diazepam**	78.6 ± 2.44***	8.4 ± 0.37***	210.4 ± 2.44***	9.2 ± 0.86***
**APSME‐200**	38.4 ± 2.22***	5.2 ± 0.37***	248.6 ± 2.44***	13.4 ±0 .58*
**APSME‐400**	61.8 ± 1.86***	7.0 ± 0.32***	224.2 ± 3.14***	11.0 ± 0.55**

*Note*: Values are Mean ± SEM, (*n* = 5); *p < 0.05, **p < 0.01 and ***p < 0.001 as compared to vehicle control (one‐way ANOVA followed by Dunnett's test). APSME = Methanolic extract of *A. papillosa* stem.

#### 3.2.2 Light Dark Test

**FIGURE 2:**
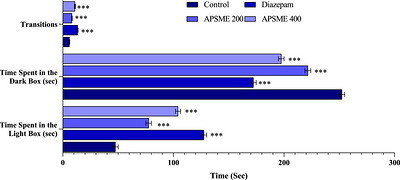
Effects of the methanolic extract of *A. papillosa* stem were evaluated using the Light–Dark Box test. All results are expressed as mean ± SEM (*n* = 5), with ***p < 0.001 considered statistically significant. APSME refers to the methanolic extract of *A. papillosa* stem.

In the Light‐Dark Box Test, administration of APSME at 200 and 400 mg/kg significantly prolonged light box exploration time compared to controls, supporting its anxiolytic efficacy (*p* < 0.001). Similarly, the standard medication substantially increased the time in light and transitions while reducing the duration in dark produced a significant (*p* < 0.001) outcome (Figure 2).

### 3.3 Sedative Activity

#### 3.3.1 Open Field Test

**FIGURE 3:**
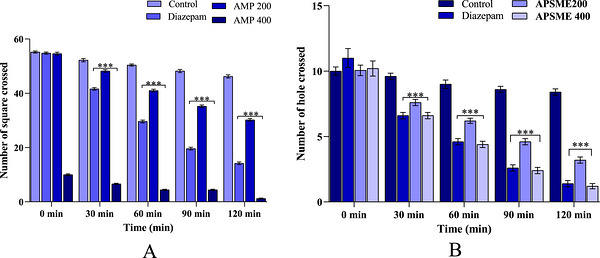
Sedative activity of the methanolic extract of *A. papillosa* stem was evaluated using the open‐field test (A) and the hole cross test (B). Results are expressed as mean ± SEM (*n* = 5), with statistical significance indicated as *p < 0.05, **p < 0.01 and ***p < 0.001 compared to the vehicle control, analysed using one‐way analysis of variance (ANOVA) followed by Dunnett's test. APSME refers to the methanolic extract of *A. papillosa* stem.

In the open‐field test, APSME produced a marked decrease in locomotor activity. At a dose of 200 mg/kg, it significantly reduced movement (*p* < 0.001), while the 400 mg/kg dose showed an even greater reduction. This indicates a clear dose‐dependent sedative effect, with outcomes comparable to the standard drug diazepam (Figure 3A).

#### 3.3.2 Hole Cross Test

From 0 min to 120 min, the conventional medication diazepam dramatically reduced locomotor activity. Additionally, APSME significantly decreased activity; at 120 min, the number of holes traversed decreased at 200 mg/kg. Notably, the 400 mg/kg dose exhibited the strongest dose‐dependent sedative effect (*p* < 0.001), with results comparable to the standard drug (Figure 3B).

### 3.4 Anti‐inflammatory Activity

#### 3.4.1 Carrageenan‐Induced Paw Oedema

In the carrageenan‐induced paw oedema model, APSME showed significant anti‐inflammatory activity when compared with the standard group. Both 200 mg/kg and 400 mg/kg doses significantly reduced paw thickness at 2, 3 and 4 h (*p* < 0.001). The 200 mg/kg dose produced a moderate but significant reduction in oedema (**p < 0.01), with 73.08% inhibition at 4 h. In contrast, the 400 mg/kg dose exhibited the highest anti‐inflammatory effect (80.77%), comparable to the standard drug (Table 3).

**TABLE 3:** Anti‐inflammatory effect of the methanolic extract of *A. papillosa* stem on carrageenan‐induced paw oedema.

Treatment (mg/kg)	Pre‐injection mean paw thickness (mm)	Post‐injection mean paw thickness (mm)			
		1 h	2 h	3 h	4 h
Control	2.55 ± 0.05	3.46 ± 0.07	3.20 ± 0.07	3.01 ± 0.07	2.81 ± 0.06
Diclofenac	2.62 ± 0.03	3.43 ± 0.03 (10.99%)	2.70 ± 0.02*** (87.69%)	2.60 ± 0.01*** (86.96%)	2.64 ± 0.01*** (92.31%)
APSME‐200	2.58 ± 0.03	3.42 ± 0.02 (7.69%)	2.95 ± 0.02** (43.08%)	2.80 ± 0.02** (52.17%)	2.65 ± 0.02** (73.08%)
APSME‐400	2.64 ± 0.02	3.45 ± 0.02 (10.99%)	2.75 ± 0.02*** (83.08%)	2.60 ± 0.01*** (84.78%)	2.69 ± 0.01*** (80.77%)

*Note*: Values are Mean ± SEM, (*n* = 5); *p < 0.05, **p < 0.01 and ***p < 0.001 as compared to vehicle control (one‐way ANOVA followed by Dunnett's test). APSME = Methanolic extract of *A. papillosa* stem.

### 3.5 Antipyretic Activity

#### 3.5.1 Brewer's Yeast‐Induced Pyrexia

APSME had a substantial antipyretic effect, where the 200 mg/kg dosage lowered temperature at 5 h (*p* < 0.001). In comparison to control, APSME 400 mg/kg significantly reduced the temperature at 5 h (*p* < 0.001). The raised temperature was dramatically lowered by the paracetamol, suggesting significant (*p* < 0.001) antipyretic action. At different time intervals, 200 mg/kg and 400 mg/kg doses show a reduction of pyrexia; the highest percentage of reduction of pyrexia is 75% at a 400 mg/kg dose (Table 4).

**TABLE 4:** Effect of the methanolic extract of *A. papillosa* stem on yeast‐induced pyrexia.

Treatment	1 h	2 h	3 h	4 h	5 h
Control	97.04 ± 0.05	101.04 ± 0.05	100.76 ± 0.05	100.54 ± 0.05	100.24 ± 0.05
Paracetamol	97.02 ± 0.06	100.34 ± 0.05*** (17.50%)	99.16 ± 0.05*** (47.00%)	98.36 ± 0.05*** (67.00%)	97.64 ± 0.05*** (85.00%)
APSME‐200	96.96 ± 0.05	100.24 ± 0.05*** (20.00%)	99.72 ± 0.04*** (33.00%)	99.24 ± 0.05*** (45.00%)	98.74 ± 0.05*** (57.00%)
APSME‐400	97.04 ± 0.05	100.3 ± 0.03*** (18.50%)	99.36 ± 0.05*** (42.00%)	98.64 ± 0.05*** (60.00%)	98.04 ± 0.05*** (75.00%)

*Note*: Values are Mean ± SEM, (*n* = 5); *p < 0.05, **p < 0.01 and ***p < 0.001 as compared to vehicle control (one‐way ANOVA followed by Dunnett's test). APSME = Methanolic extract of *A. papillosa* stem.

### 3.6 In Silico Study

#### 3.6.1 In Silico Molecular Docking Study

The docking analysis revealed different binding affinities of the APSME‐derived phytochemicals toward the selected targets associated with anxiety, sedation, inflammation and fever (Table 5).

**TABLE 5:** Binding affinities (kcal/mol) of phytochemicals identified from the methanol extract of the stem of *A. papillosa* (APSME) against important molecular targets associated with anxiety, sedation, inflammation and fever.

SL. No	Phytochemical	PubChem ID	Anxiolytic	Sedative	Anti‐inflammatory	Antipyretic
			2Z5X	6HUP	5IKR	3DWW
1	*N*‐Methyl‐4‐pyridinamine	123098	−4.6	−5	−4.7	−3.4
2	Dimethyl sulfone	6213	−2.8	−3.2	−3	−2.7
3	p‐Cresol	2879	−5.5	−5.7	−5.4	−3.9
4	2‐Acetyl‐5‐methylfuran	14514	−5.5	−5.6	−5.6	−4.3
5	Ethanone, 1‐(2,4,6‐trihydroxyphenyl)‐	68073	−6.1	−6.9	−6.2	−4.5
6	Benzeneacetic acid, 2‐phenylethyl ester	7601	−8.8	−8.8	−7.6	−4.4
7	2‐Butyn‐1‐al diethyl acetal	137721	−4.8	−5.2	−4.9	−3.4
8	6‐Ethyl‐5,6‐dihydro‐2H‐pyran‐2‐one	12213801	−5.6	−5.9	−5.7	−4
9	1,3,5‐Cycloheptatriene, 7‐ethyl‐	561243	−5.9	−6.8	−6.2	−4.1
10	Phenol, 4‐(methoxymethyl)‐	79310	−5.5	−6	−5.8	−4
11	Ethanone, 1‐(2‐hydroxy‐5‐methylphenyl)‐	15068	−6.7	−6.9	−6.4	−4.5
12	1—(4‐Hydroxyphenyl)propane‐1,2‐diol	75411975	−6.5	−6.7	−5.9	−4.5
13	Homovanillyl alcohol	16928	−6	−6.5	−6	−4.4
14	2,4‐Di‐tert‐butylphenol	7311	−8.1	−7.3	−6	−4.1
15	Methyl 3‐(4‐hydroxy‐3‐methoxyphenyl)propanoate	523498	−6.7	−7.1	−6.6	−4.4
16	Methyl tetradecanoate	31284	−6.5	−6.8	−6.2	−3.2
17	Pentadecanoic acid, methyl ester	23518	−6.2	−6.7	−6.1	−3.4
18	Hexadecanoic acid, methyl ester	8181	−6.2	−6.5	−6.1	−3.5
19	Heptadecanoic acid, methyl ester	15609	−6.3	−6.8	−5.9	−3.4
20	9,12‐Octadecadienoic acid (Z,Z)‐, methyl ester	5284421	−6.5	−7.1	−6.5	−3.6
21	11‐Octadecenoic acid, methyl ester	5364432	−6.6	−6.8	−6.2	−3.6
22	9‐Octadecenoic acid (Z)‐, methyl ester	5364509	−6	−6.7	−6.1	−3.4
23	Methyl stearate	8201	−5.9	−6.6	−6.3	−3.5
24	Nonadecanoic acid, methyl ester	15610	−6.5	−6.6	−6.5	−3.5
25	Eicosanoic acid, methyl ester	14259	−6.1	−6.8	−6.2	−3.5
26	Docosanoic acid, methyl ester	13584	−5.7	−6.5	−6.2	−3.3
27	Standard	3016; 3033; 1983	−4.2	−9.5	−8.1	−4

#### 3.6.2 Docking Study for Anxiolytic Activity

**FIGURE 4:**
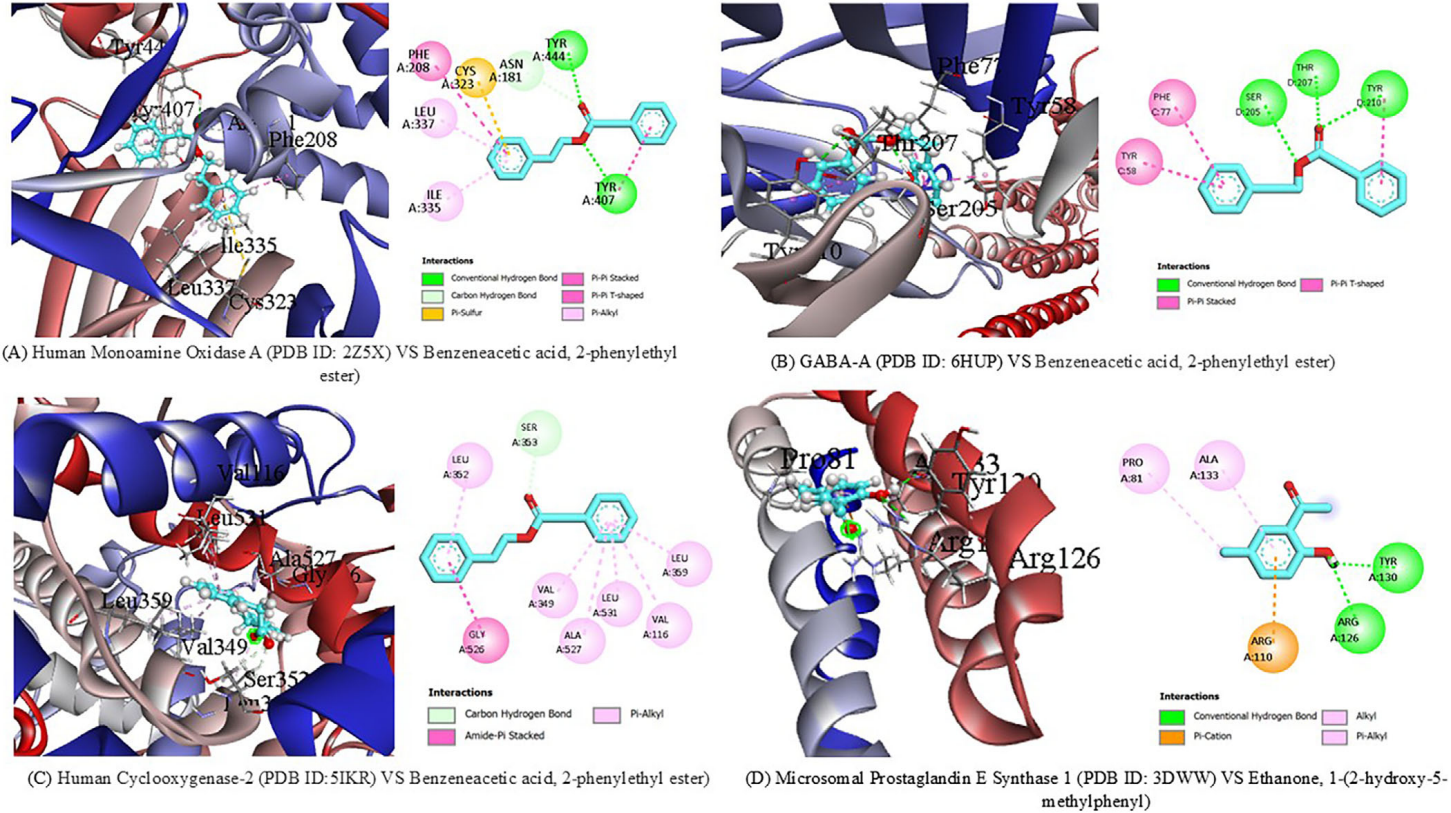
Two‐ and three‐dimensional representations of the highest‐scoring docked phytochemicals derived from the methanolic stem extract of *A. papillosa* were generated against key protein targets associated with anxiety, sedation, inflammation and fever.

Molecular docking studies were conducted with the phytochemicals obtained from APSME in order to examine their anxiolytic potential. The outcome from these studies evidenced that the binding affinities of the various phytochemicals ranged between ‐2.8 and ‐8.8 kcal/mol. Benzeneacetic acid, 2‐phenylethyl ester, had the greatest binding affinity (‐8.8 kcal/mol), which was higher than that of the standard drug, diazepam (‐4.2 kcal/mol). Additionally, benzeneacetic acid, 2‐phenylethyl ester, formed two conventional hydrogen bonds with Tyr‐407 and Tyr‐444, a carbon‐hydrogen bond with Asn‐181 and multiple hydrophobic interactions, such as π‐π stacked interactions with Tyr‐407, π‐π T‐shaped interactions with Phe‐208, π‐alkyl interactions with Ile‐335 and Leu‐337 and π‐sulphur interactions with Cys‐323, thus providing strong evidence to support the anxiolytic potential of this compound based upon its ability to bind well to the target protein (Figure 4A).

#### 3.6.3 Docking Study for Sedative Activity

Molecular docking was used to assess the sedative effects of phytochemicals from APSME on the GABA‐A receptor (PDB ID: 6HUP), shown in Figure 4B. The phytochemical with the highest binding affinity was benzeneacetic acid, 2‐phenylethyl ester (−8.8 kcal/mol), compared to the reference sedative drug, diazepam (−9.5 kcal/mol). The above phytochemical made three conventional hydrogen bonds (to Ser‐205, Thr‐207 and Tyr‐210), formed two π–π stacked interactions (with Tyr‐58 and Tyr‐210) and formed one π–π T‐shaped interaction (with Phe‐77). These multiple interactions suggest a strong binding affinity toward the target receptor, indicating its potential to exhibit sedative effects.

#### 3.6.4 Docking Study for Anti‐inflammatory Activity

The anti‐inflammatory potential of the compounds shown in Figure 4C was evaluated by analysing their non‐bonding interactions with amino acid residues of human COX‐2 (PDB ID: 5IKR). All selected molecules were further subjected to molecular docking against their respective target proteins. The compounds with the strongest predicted binding affinity to the target proteins were benzeneacetic acid and diclofenac, with predicted binding affinities of ‐6.4 and ‐7.6 kcal/mol, respectively. The compounds created one hydrogen bond with Ser‐353 and eight hydrophobic interactions, including two hydrophobic interactions with amide‐π stacking bonds involving Gly‐526 and Ala‐527, and the remaining six hydrophobic interactions involved π‐alkyl interactions with Leu‐352, Val‐116, Val‐349, Ala‐527, Leu‐359 and Leu‐531.

#### 3.6.5 Docking Study for Antipyretic Activity

Molecular docking studies on phytochemical compounds from APSME were performed to assess their ability to inhibit fever by binding to Microsomal Prostaglandin E Synthase‐1 (PDB ID: 3DWW). The compounds yielded a range of calculated binding affinities of ‐2.7 to ‐4.5 kcal/mol. For three of these phytochemicals, ethenone 1‐(2‐hydroxy‐5‐methylphenyl)‐, ethenone 1‐(2,4,6‐trihydroxyphenyl)‐ and 1‐(4‐hydroxyphenyl)‐propane‐1,2‐diol, they all have an overall docking score of ‐4.5 kcal/mol, similar to paracetamol (‐4 kcal/mol) and stronger interactions with the target protein. The strongest interaction was seen with Ethenone 1‐(2‐hydroxy‐5‐methylphenyl), where conventional hydrogen bonds between this phytochemical and the amino acid residues of Tyr‐130 and Arg‐126, a π‐cation interaction with Arg‐110 and several hydrophobic interactions (Pro‐81 and Ala‐133) were observed (Figure 4D).

#### 3.6.6 PASS Prediction

The APSME compounds chosen for this research demonstrated both anti‐anxiety/sedation properties as well as the ability to reduce inflammation and fever. The PASS computer prediction program showed favourable biological activity probabilities (Pa > Pi) compared to all other compounds that have been previously studied (Table 6).

**TABLE 6:** Biological activity of the methanolic extract of *A. papillosa* stem through PASS prediction analysis.

Phytochemical	Anxiolytic		Sedative		Anti‐inflammatory		Antipyretic	
	Pa	Pi	Pa	Pi	Pa	Pi	Pa	Pi
N‐Methyl‐4‐pyridinamine	0.348	0.075	0.341	0.03	0.3	0.16	0.288	0.05
Dimethyl sulfone	—	—	—	—	—	—	—	—
p‐Cresol	0.706	0.004	0.608	0.005	0.456	0.071	0.451	0.02
2‐Acetyl‐5‐methylfuran	0.448	0.032	0.377	0.024	0.645	0.024	0.212	0.103
Ethenone, 1‐(2,4,6‐trihydroxyphenyl)‐	0.636	0.007	0.539	0.009	0.661	0.021	0.496	0.014
Benzeneacetic acid, 2‐phenylethyl ester	0.456	0.03	0.42	0.019	0.674	0.019	0.428	0.022
2‐Butyn‐1‐al diethyl acetal	0.556	0.013	0.269	0.047	0.318	0.058	0.218	0.098
6‐Ethyl‐5,6‐dihydro‐2H‐pyran‐2‐one	0.621	0.008	0.291	0.041	0.476	0.064	0.375	0.029
1,3,5‐Cycloheptatriene, 7‐ethyl‐	0.58	0.011	0.484	0.012	0.397	0.024	0.256	0.067
Phenol, 4‐(methoxymethyl)‐	0.687	0.005	0.396	0.021	0.573	0.004	0.34	0.035
Ethanone, 1‐(2‐hydroxy‐5‐methylphenyl)‐	0.538	0.015	0.367	0.025	0.585	0.035	0.648	0.005
1‐(4‐Hydroxyphenyl)propane‐1,2‐diol	0.629	0.007	0.344	0.029	0.655	0.003	0.233	0.085
Homovanillyl alcohol	0.482	0.024	0.241	0.057	0.445	0.005	0.359	0.031
2,4‐Di‐tert‐butylphenol	0.555	0.013	0.438	0.017	0.762	0.009	0.468	0.017
Methyl 3‐(4‐hydroxy‐3‐methoxyphenyl)propanoate	0.645	0.006	0.241	0.057	0.551	0.005	0.477	0.016
Methyl tetradecanoate	0.82	0.003	0.366	0.026	0.758	0.002	0.323	0.038
Pentadecanoic acid, methyl ester	0.82	0.003	0.366	0.026	0.758	0.002	0.323	0.038
Hexadecanoic acid, methyl ester	0.82	0.003	0.366	0.026	0.758	0.002	0.323	0.038
Heptadecanoic acid, methyl ester	0.82	0.003	0.366	0.026	0.758	0.002	0.323	0.038
9,12‐Octadecadienoic acid (Z,Z)‐, methyl ester	0.749	0.004	0.299	0.039	0.728	0.013	0.255	0.068
11‐Octadecenoic acid, methyl ester	0.782	0.003	0.355	0.031	0.717	0.002	0.294	0.047
9‐Octadecenoic acid (Z)‐, methyl ester	0.782	0.003	0.355	0.031	0.717	0.002	0.294	0.047
Methyl stearate	0.82	0.003	0.366	0.026	0.758	0.002	0.323	0.038
Nonadecanoic acid, methyl ester	0.82	0.003	0.366	0.026	0.758	0.002	0.323	0.038
Eicosanoic acid, methyl ester	0.82	0.003	0.366	0.026	0.758	0.002	0.323	0.038
Docosanoic acid, methyl ester	0.82	0.003	0.366	0.026	0.758	0.002	0.323	0.038

## 4 Discussion

The use of plant medicine has increased substantially in recent decades, allowing for the development of new therapies for many chronic conditions. Among these, many orchids are used as medicinal herbs in traditional medicine throughout the world [40]. Orchids produce a variety of bioactive compounds from their various plant parts that have been shown to have widespread medicinal effects, including antipyretics (fever reducers), leuko‐stimulants (molecules that stimulate the production of white blood cells), treatment of ocular conditions (eye infections), relief from fatigue and headaches and anticancer actions [41]. In this study, 26 bioactive compounds from APSME were characterised with the GC‐MS method and in vivo animal experiments and computational modelling were used to assess their biological activity.

EPM offers a probable measure of rodent/anxiolytic activity by providing rodents with two types of environments: closed‐arm (i.e. traditional shelter) and open‐arm (exposed). When anxious, rodents will prefer to use closed arms, but an increase in exploration of open arms suggests reduced levels of anxiety [42]. APSME produced a significant, dose‐dependent increase in time spent in the open arms compared with the control group (38.4 ± 2.22 s and 61.8 ± 2.86 s at 200 and 400 mg/kg, respectively), indicating an anxiolytic effect. Correspondingly, the time spent in the closed arms decreased with increasing dose. Although the standard drug diazepam produced the greatest level of anxiety reduction, APSME still produced considerable anxiolytic activity. In addition, the light–dark box test assesses anxiety by quantifying their avoidance of lighted areas [43]. The standard drug significantly increased the time spent in the light compartment (127.6 ± 2.5 s) and produced 13.4 ± 0.51 transitions, along with a corresponding reduction in time spent in the dark compartment (172.4 ± 2.5 s). Doses of APSME produced similar but dose‐dependent effects for light and dark; for example, at 200 mg/kg, the TST in the light compartment was 77.6 ± 2.5 s, and the number of transitions was 8.2 ± 0.37, while at 400 mg/kg, the TST increased to 104.2 ± 2.29 s with 11.0 ± 0.45 transitions. A reduction in the time spent in the dark compartment was observed in rodents treated with APSME at both doses.

Diazepam, a benzodiazepine, works as a depressant on our central nervous system by binding with the GABA receptor complex, which produces a calming/sedative effect. The benzodiazepine's depressing effect also reduces CNS excitation, extends the duration of barbiturate‐induced sleep and decreases the amount of exploration. On behavioural models such as open field & hole cross tests (HCTs), sedative agents decrease locomotor activity, giving the opposite of alertness: increased levels of sedation [44]. On both tests, APSME decreased locomotor activity with significant levels demonstrating a sedative impact. In the open‐field test, animals treated with APSME at a dose of 200 mg/kg showed a marked reduction in locomotor activity, with movements decreasing from 54.6 ± 0.5 to 30.2 ± 0.5 at 120 min post‐administration. A 400 mg/kg APSME exhibited a larger dose‐related response, with animals making only 10.0 ± 0.32 movements, which was further reduced to only 1.2 ± 0.20. Alternatively, using an HCT, animals treated with diazepam exhibited a marked decrease in activity after administration at 120 min, with only 10.0 ± 0.32 movements, reduced to only 1.4 ± 0.24. Also, when evaluating APSME, at the 200 mg dose, the number of hole crossings was reduced compared to the 400 mg dose, with holes crossed from 3.2 ± 0.20 to 1.2 ± 0.20. These numbers demonstrate that APSME has strong sedative activity comparable to established sedative agents.

The carrageenan‐induced paw oedema model is widely employed to assess anti‐inflammatory activity, as the development of oedema is driven by key inflammatory mediators, including serotonin, histamine and prostaglandins [45]. The paw oedema induced by APSME was significantly reduced and demonstrated strong anti‐inflammatory activity. The activity is likely due to the presence of steroids and glycosides in this extract that may inhibit the release or function of inflammatory mediators [45]. Therefore, the evidence presented here suggests that APSME has some potential therapeutic benefit in managing acute inflammatory conditions.

In a yeast‐induced fever study, mice that received a subcutaneous 30% suspension of brewer's yeast had markedly elevated rectal temperatures after one and a half hours (18 h post‐administration), due to pro‐inflammatory cytokines released by the yeast and acting in the circulation to stimulate production of PGE2 by the hypothalamic thermoregulatory centre and surrounding tissues [46]. All the tested concentrations of APSME (Table 3) reduced rectal temperatures significantly relative to the negative control group in a dose‐dependent manner; the effects at both doses approximated those of paracetamol, the standard antipyretic treatment.

Computational methods, especially molecular docking, generate valuable complementarity to data produced from in vivo experiments, including estimates of how the bioactive compounds bind to their target proteins. Molecular docking is an excellent tool for identifying potential lead compounds from those natural constituents that were individualised using GC‐MS, providing insight into how tightly the lead compound will bind to its target (binding affinity) and if it is druggable (pharmacological potential), as well as providing insight into possible mechanisms of action [47, 48].

Molecular docking analysis of phytochemicals from APSME has shown that these compounds likely have beneficial binding to several protein targets associated with sedative, anxiolytic, anti‐inflammatory and antipyretic drugs. Specifically, benzeneacetic acid, 2‐phenylethyl ester, showed the highest predicted binding interactions with the protein's monoamine oxidase A and GABA‐A at −8.8 kcal/mol each. Thus, it is predicted that this compound may exert anxiolytic and sedative‐like properties. For potential anti‐inflammatory activity, both benzeneacetic acid and diclofenac had strong predicted interactions with COX‐2 (predicted binding affinities: −6.4 and −7.6 kcal/mol, respectively). In addition, ethenone (1‐(2‐hydroxy‐5‐methylphenyl)‐) had the highest predicted binding affinity for microsomal prostaglandin E synthase at −4.5 kcal/mol and therefore may exert antipyretic activity. Overall, APSME phytochemicals are predicted to have significant binding to multiple therapeutic targets, thereby providing preliminary evidence for their therapeutic value in the treatment of anxiety/sedation, inflammation, and fever. The PASS prediction analysis, together with molecular docking findings and previous literature reports, supports the hypothesis that APSME phytochemicals may be effective in treating conditions related to anxiety/sedation, inflammation and fever.

## 5 Conclusion

The APSME was assessed for its ability to produce an anxiolytic effect, sedative effect, anti‐inflammatory effect and antipyretic effect in mice by means of both behavioural and physiological testing. Molecular docking studies provided evidence of strong molecular interactions between major active ingredients of *A. papillosa* extract (e.g., Benzeneacetic acid 2‐phenylethyl ester and Ethanone 1‐(2‐hydroxy‐5‐methylphenyl)‐) and targeted proteins. PASS prediction analysis corroborated the therapeutic candidate status of the active ingredients from *A. papillosa*. The present findings indicate that *A. papillosa* may represent an attractive natural product source for generating new therapeutic candidates for treating anxiety, sedation, inflammation and fever.

## Author Contributions

**Qurratul Ain Sadia**: conceptualisation, methodology, software, visualisation and writing – original draft. **Sadia Tamanna Tamim**: methodology, software, formal analysis, visualisation and writing – original draft. **Faruq Mohammed Tashriq**: investigation, formal analysis, software and writing – original draft. **Nasir Uddin**: investigation, formal analysis and writing – original draft. **Fatematus Zhohara Alam Hima**: investigation, formal analysis and writing – original draft. **Ulfat Sobha Surat**: investigation, formal analysis and writing – original draft. **Mily Khastagir**: investigation and formal analysis. **Muhammad Abdul Jalil**: investigation and data curation. **Md. Hossain Rasel**: investigation and data curation. **Arman Ullah Rafi**: investigation. **S. M. Moazzem Hossen**: supervision, conceptualisation, resources, project administration, funding acquisition and writing – review & editing.

## Funding

The authors have nothing to report.

## Ethics Statement

The animal experiment was approved by the Departmental Ethical Review Committee of the University of Chittagong under approval number AERB‐FBSCU‐2026‐05‐04‐01‐93379.

## Conflicts of Interest

The authors declare no conflicts of interest.

## Artificial Intelligence Use Statement

Artificial intelligence (AI) tools were used only to assist with language editing, including grammar correction, sentence improvement and enhancing the overall readability of the manuscript. AI was not used in the design of the study, data collection, data analysis, interpretation of results, preparation of figures, or development of scientific conclusions. All experimental work, analyses, interpretations and conclusions were carried out by the authors. The authors carefully reviewed and verified all AI‐assisted edits and take full responsibility for the accuracy and integrity of the manuscript.

## Supporting information




**Supporting File 1**: ansa70097‐sup‐0001‐SuppMat.docx.

## Data Availability

All data generated in this study are kept confidential; however, they can be made available by the corresponding author upon reasonable request.
