# Supplementary material for: Integrated Phytochemical Characterisation, Pharmacological Evaluation and Computational Investigation of Acampe papillosa Stem Extract
Source: Anal Sci Adv. 2026 Jul 23;7(2):e70097. doi: 10.1002/ansa.70097 (PMC13395471; doi:10.1002/ansa.70097)
Supplement: Supplementary file 1 — Supporting File 1: ansa70097‐sup‐0001‐SuppMat.docx. [file ANSA-7-e70097-s001.docx]

**MASS SPECTROMETRY of all identified compounds**


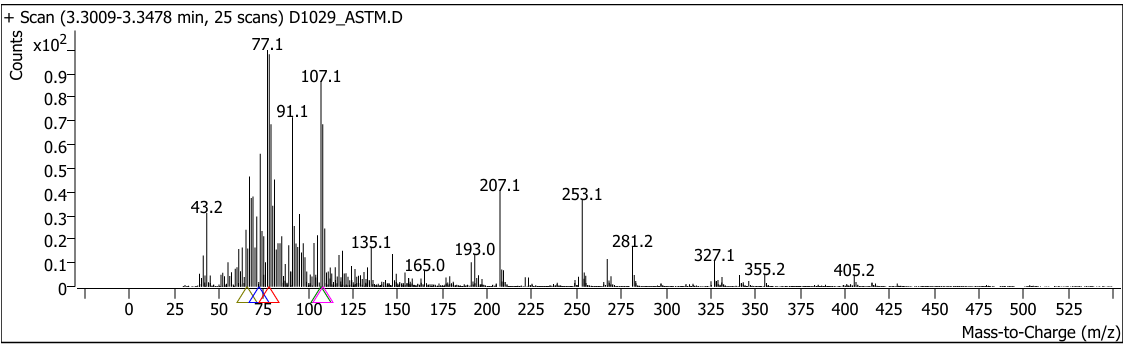


**Figure S1.** N-Methyl-4-pyridinamine


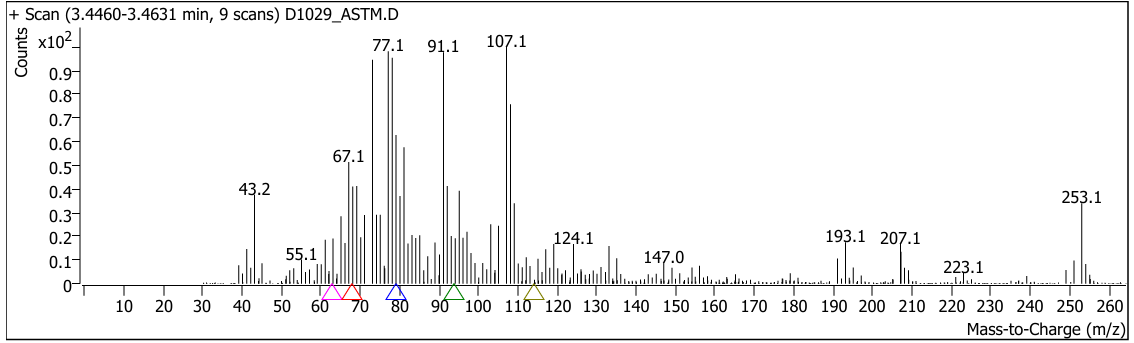


**Figure S2.** Dimethyl sulfone


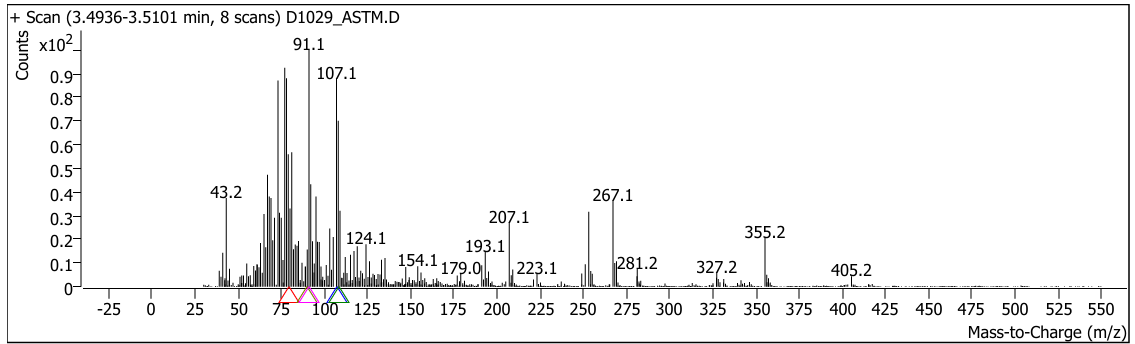


**Figure S3.** p-Cresol


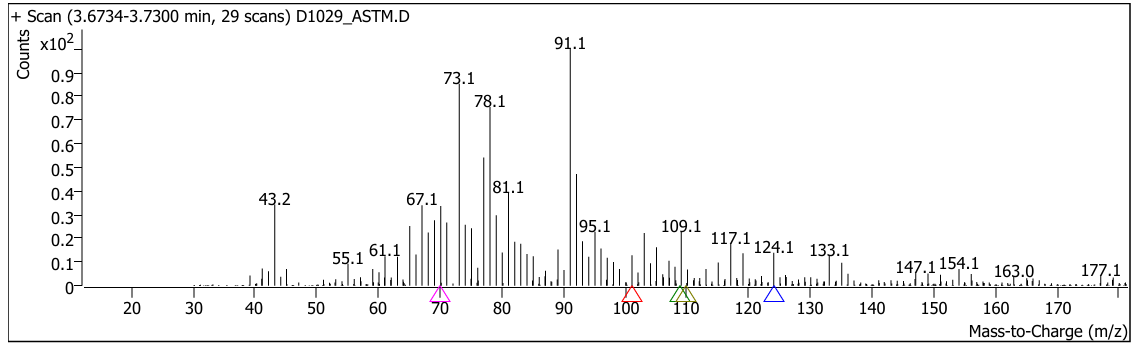


**Figure S4.** 2-Acetyl-5-methylfuran


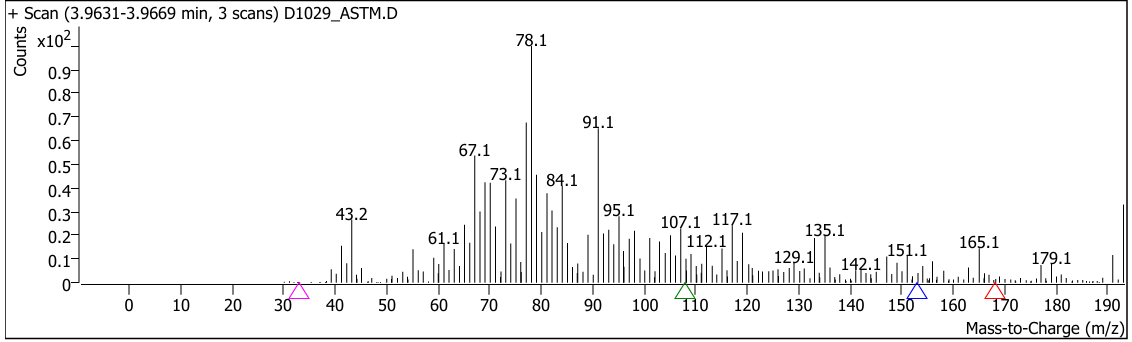


**Figure S5.** Ethanone, 1-(2,4,6-trihydroxyphenyl)-


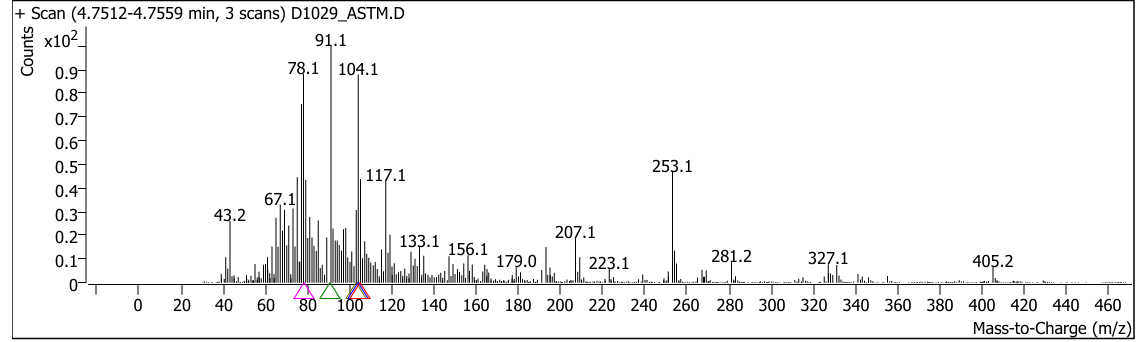


**Figure S6.** Benzeneacetic acid, 2-phenylethyl ester


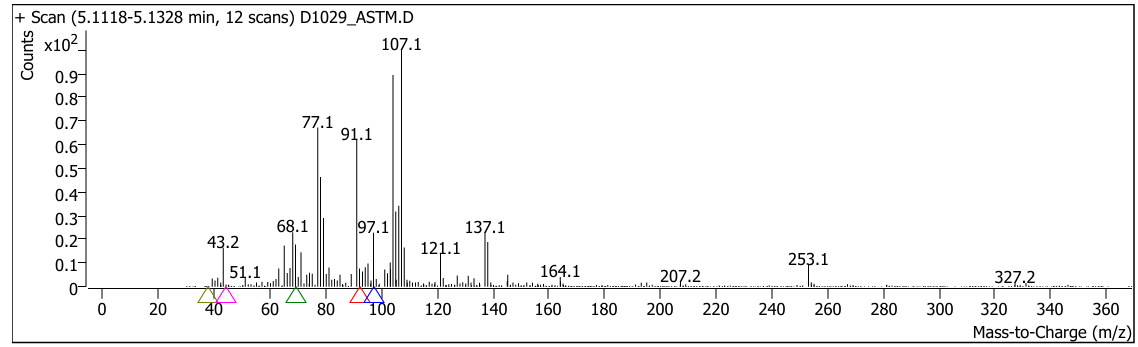


**Figure S7.** 2-Butyn-1-al diethyl acetal


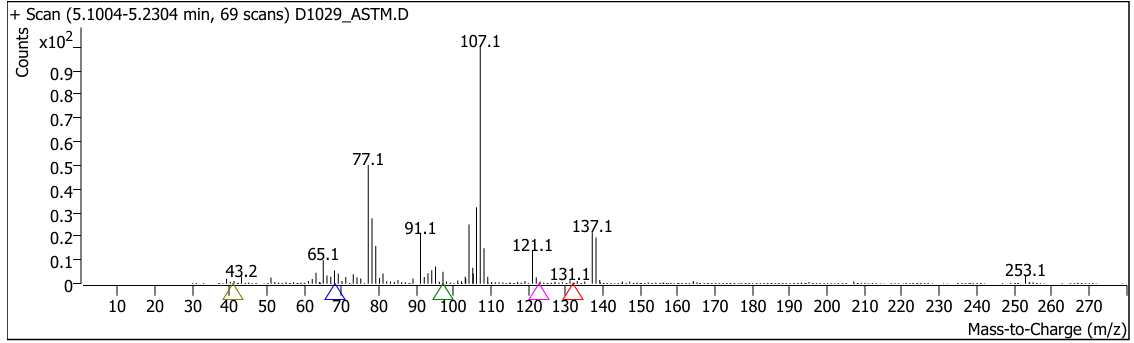


**Figure S8.** 6-Ethyl-5,6-dihydro-2H-pyran-2-one


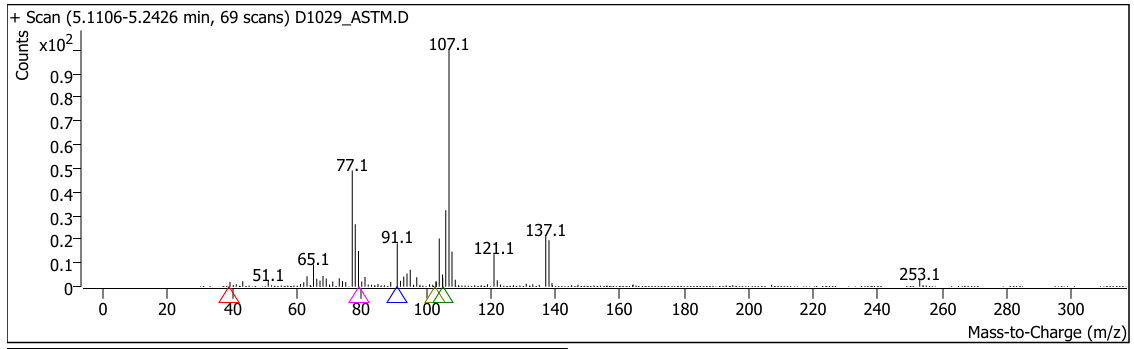


**Figure S9.** 1,3,5-Cycloheptatriene, 7-ethyl-


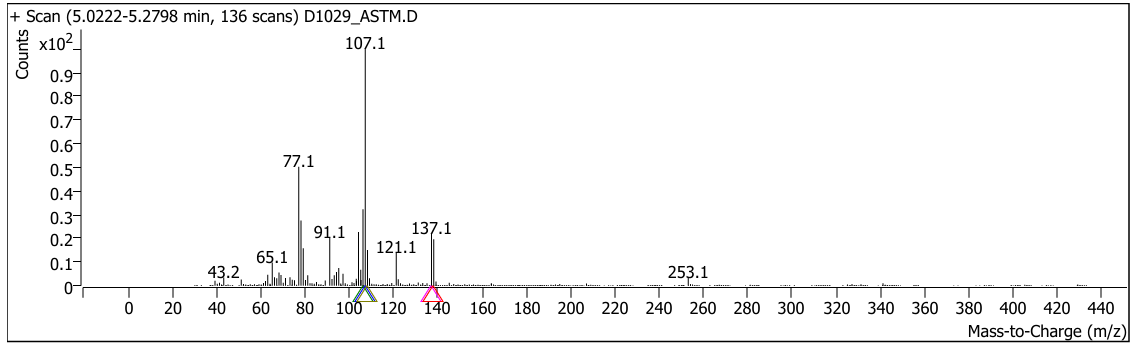


**Figure S10.** Phenol, 4-(methoxymethyl)-


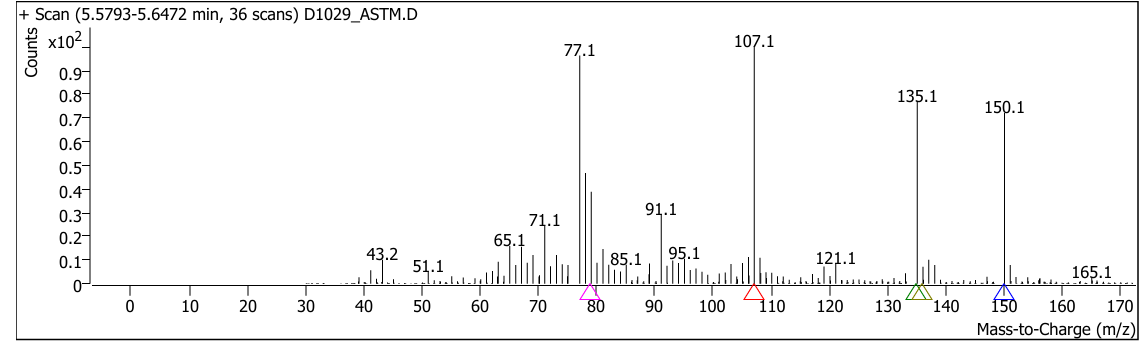


**Figure S11.** Ethanone, 1-(2-hydroxy-5-methylphenyl)-


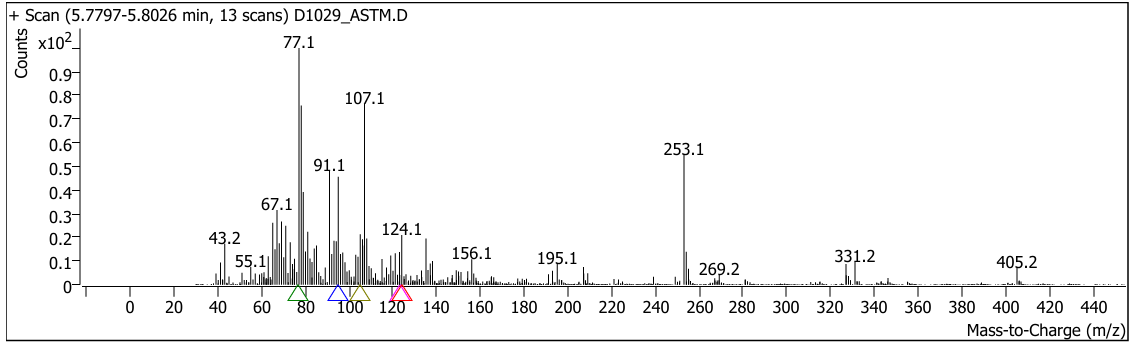


**Figure S12.** 1-(4-Hydroxyphenyl)propane-1,2-diol


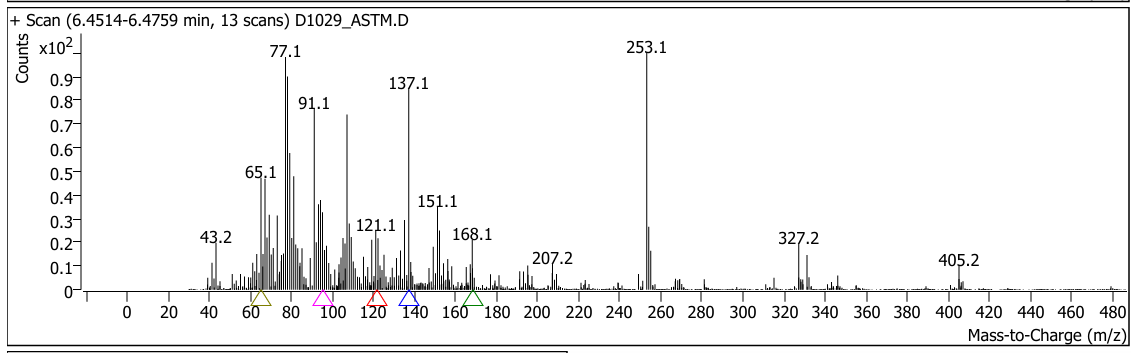


**Figure S13.** Homovanillyl alcohol


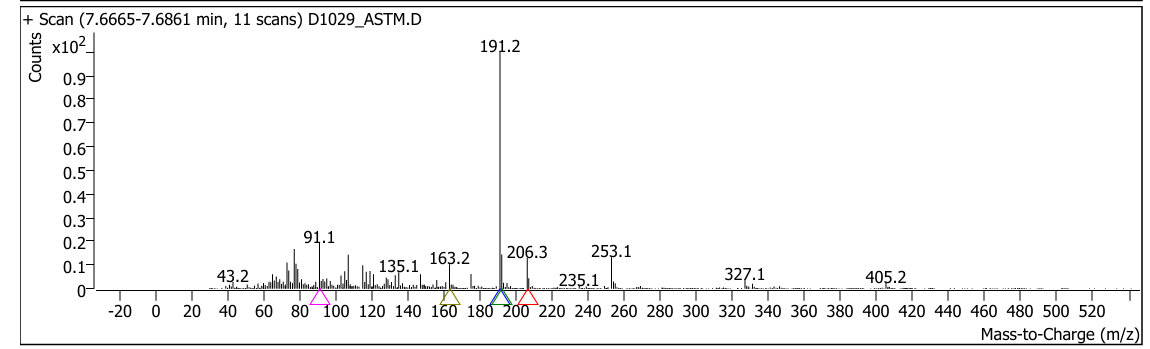

**Figure S14.** 2,4-Di-tert-butylphenol


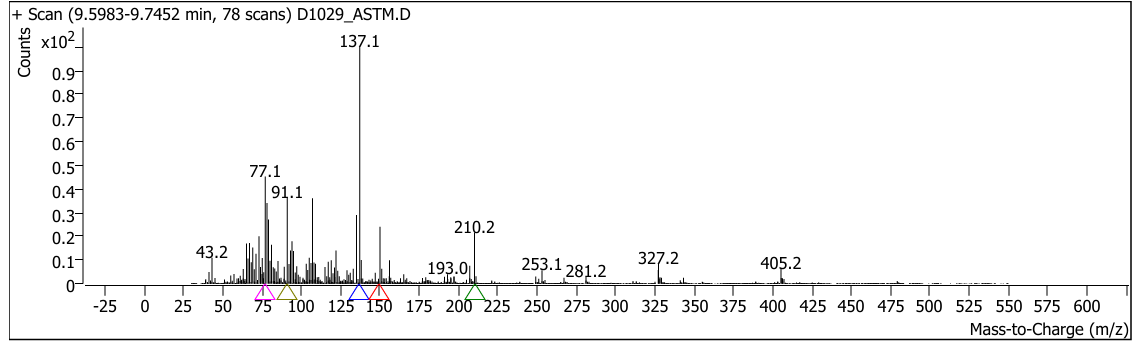


**Figure S15.** Methyl 3-(4-hydroxy-3-methoxyphenyl)propanoate


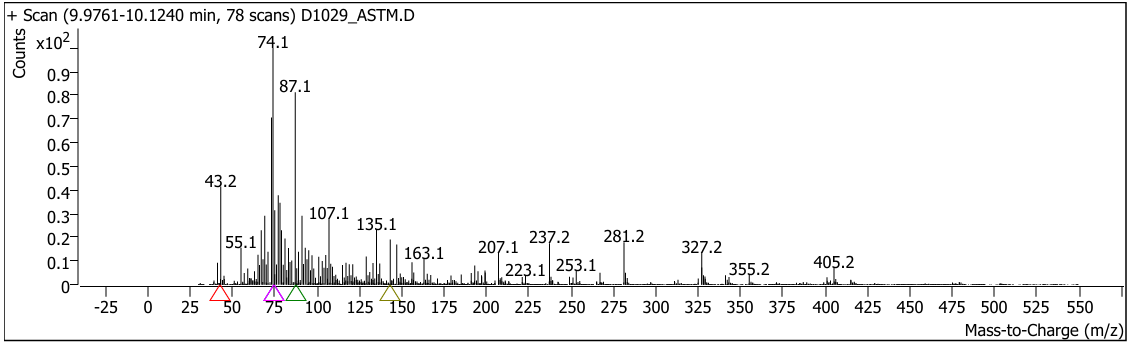


**Figure S16.** Methyl tetradecanoate


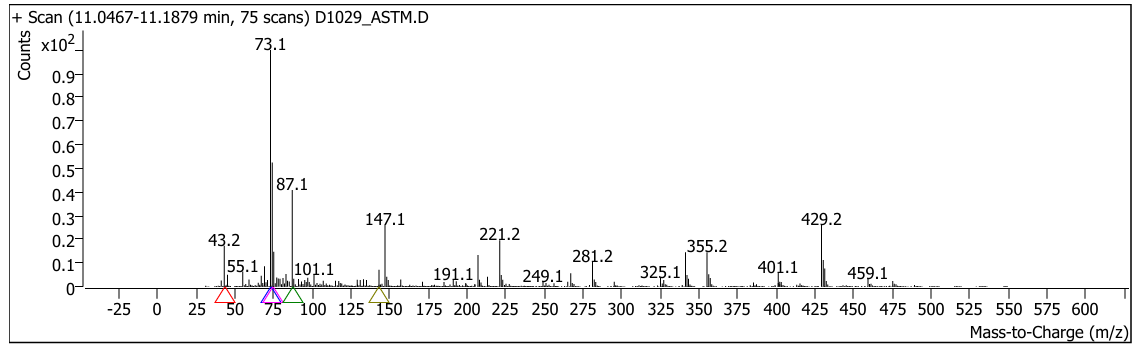


**Figure S17.** Pentadecanoic acid, methyl ester


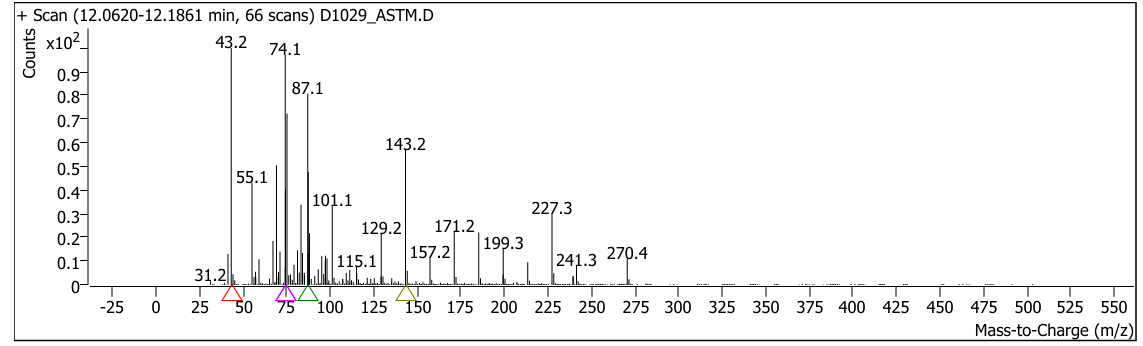


**Figure S18.** Hexadecanoic acid, methyl ester


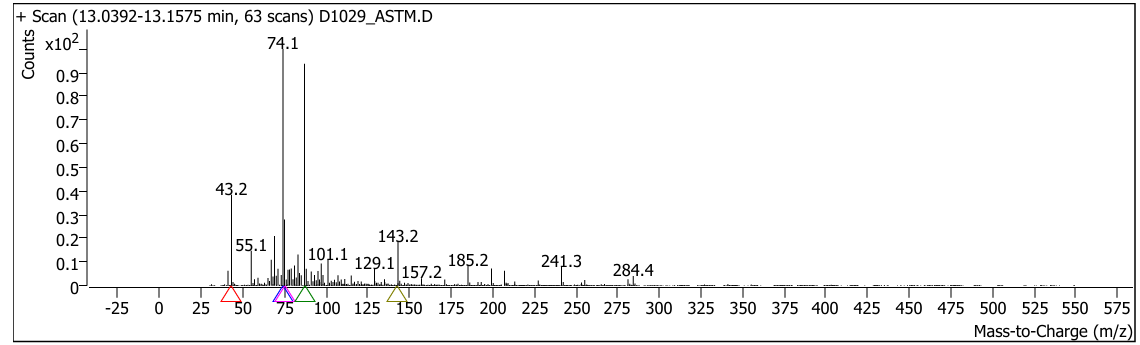


**Figure S19.** Heptadecanoic acid, methyl ester


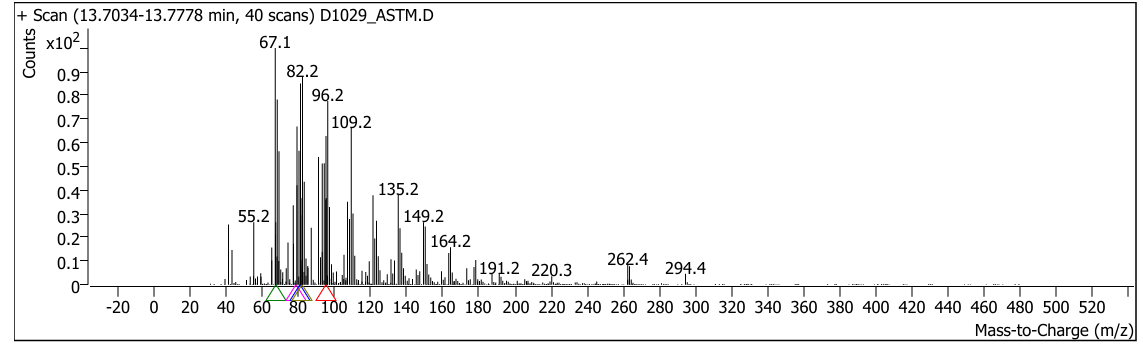


**Figure S20.** 9,12-Octadecadienoic acid (Z, Z)-, methyl ester


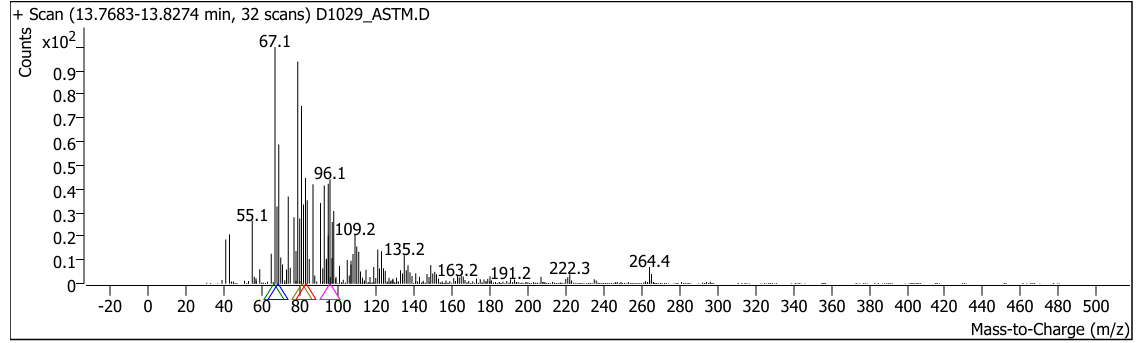


**Figure S21.** 11-Octadecenoic acid, methyl ester


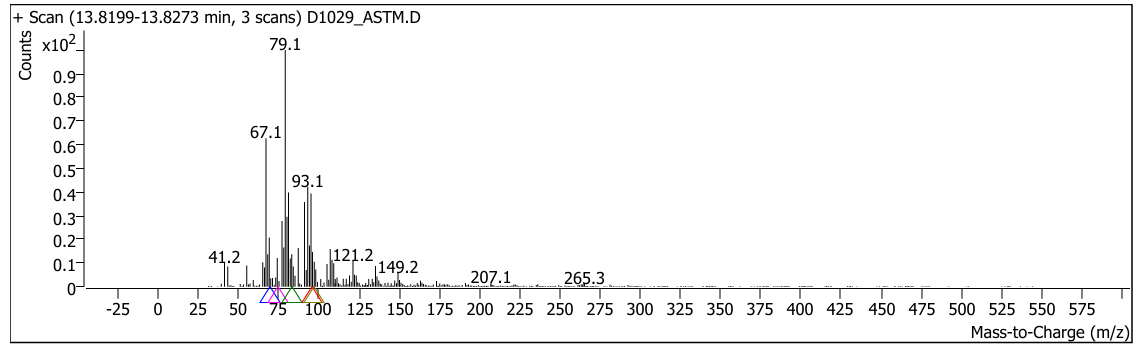


**Figure S22.** 9-Octadecenoic acid (Z)-, methyl ester


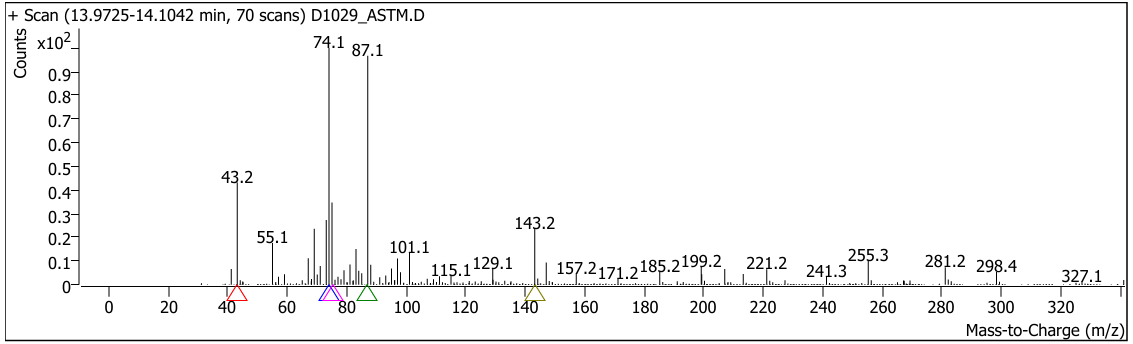


**Figure S23.** Methyl stearate


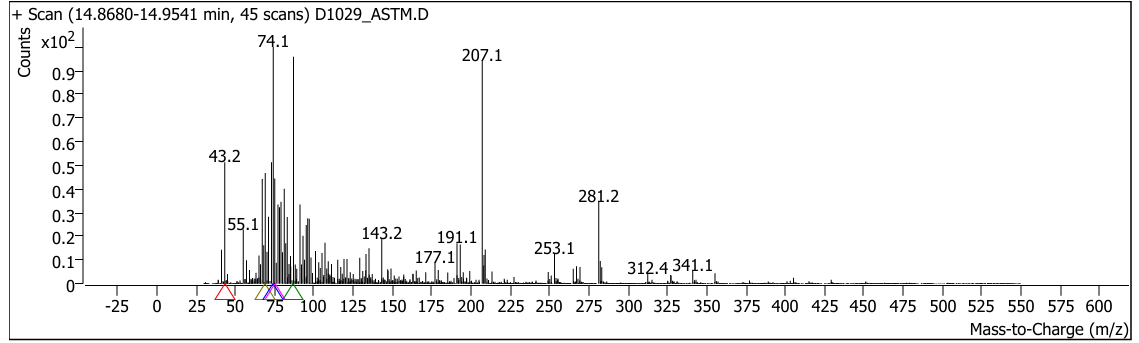


**Figure S24.** Nonadecanoic acid, methyl ester


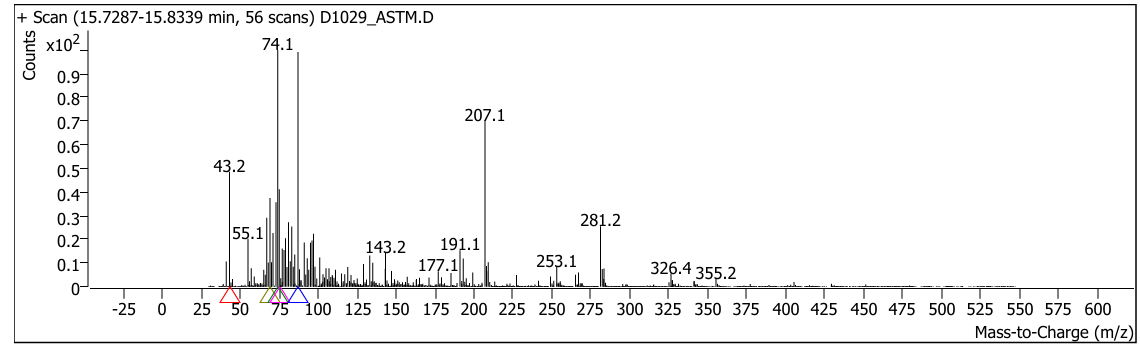


**Figure S25.** Eicosanoic acid, methyl ester


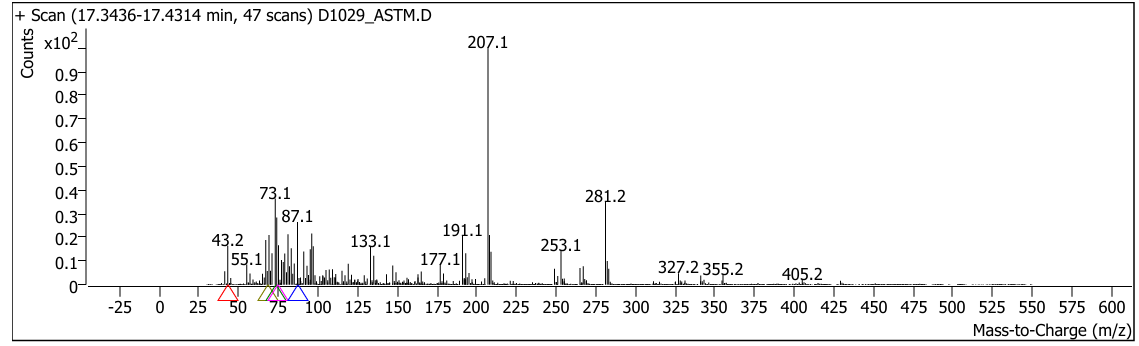


**Figure S26.** Docosanoic acid, methyl ester
